# Editorial: Reviews in bioresorbable scaffold

**DOI:** 10.3389/fcvm.2024.1437555

**Published:** 2024-06-05

**Authors:** Vikrant Rai, Juan F. Iglesias, Matthias Bossard, Khagendra Dahal, Chun Chin Chang, Mariusz Tomaniak

**Affiliations:** ^1^ Department of Translational Research, Western University of Health Sciences, Pomona, CA, United States; ^2^ Department of Cardiology, Geneva University Hospitals, Geneva, Switzerland; ^3^ Cardiology Division, Heart Center, Luzerner Kantonsspital, Luzern, Switzerland; ^4^ School of Medicine, Creighton University, Omaha, NE, United States; ^5^ Division of Cardiology, Department of Medicine, Taipei Veterans General Hospital, Taipei, Taiwan; ^6^ 1^st^ Department of Cardiology, Medical University of Warsaw, Warsaw, Poland

**Keywords:** coronary artery disease, revascularization, percutaneous coronary intervention (PCI), bioresorbable scaffolds (BRS), drug-eluting stents (DES)

**Editorial on the Research Topic** Reviews in bioresorbable scaffold

Bioresorbable scaffolds (BRS) providing mechanical support and delivering the drug post percutaneous coronary intervention (PCI) for vascular restoration are either composed of naturally occurring and synthetic biodegradable polymers or dissolving metals. BRS are anticipated to mitigate, to some extent, the issues associated with permanent metallic drug-eluting stents (DES) including polymer hypersensitivity, incomplete endothelialization, neoatherosclerosis, stent fracture, and a 2%–4% annual incidence of stent-related cardiac outcomes (1).

An optimal BRS has the advantages of progressive bioresorption, adaptive vascular remodeling, recovery of cyclic pulsatility and vasoregulation, normalization of shear stress and cyclic strain, as well as restoration of normal vascular curvature among others (2), Peng et al. However, along with various advantages, procedural technical difficulties, intraluminal scaffold dismantling, and higher early rates of scaffold thrombosis and target vessel-related myocardial infarction, (TVMI) particularly in small vessels, coexists. A study by Abellas-Sequeiros et al. (3) reported target lesion failure (TLF) of 4.7% at 12 months after magnesium-based BRS (Magmaris^TM^, Biotronik AG, Switzerland) deployment; however, a randomized controlled trial by Stone et al. (4) reported a 3% greater absolute 5-year rate of TLF after polymer-based BRS (Absorb^TM^, Abbott Vascular, USA) compared with cobalt-chromium everolimus-eluting stents and a metanalysis by Wan et al. reported significantly higher in-stent diameter stenosis within 1 year with a similar risk of target lesion revascularization and cardiac death with polymer- and magnesium-based BRS. These results warrant more studies to evaluate the pros and cons of BRS and their use in reducing the risk of adverse events and improving the clinical outcomes and the long-term prognosis in patients with ischemic events (5).

The aim of the Research Topic “*Reviews in Bioresorbable Scaffold*” was to highlight recent advances in using BRS for coronary stenting, whilst emphasizing important directions and new opportunities for future technological amendments. The presented special issue, comprising five original articles, focuses on the impact of BRS on clinical outcomes, resulting lumen inflammation and damage, the role of intravascular imaging in PCI with BRS, and the predictors for adverse outcomes with BRS.

Truong et al. reported in a single-center prospective study outcomes following PCI with Magmaris™ BRS implantation, using imaging (intravascular ultrasound) guidance. They highlight in a real-world practice, including a 60.3% of all lesions within the LAD mainly type B1 lesions (51.7%), that this strategy is safe and effective option in coronary artery disease patients. The Magmaris™ BRS was used in 60 patients with no adverse in-hospital events, one patient each for myocardial infarction, stroke event, non-target-lesion revascularization, in-stent thrombosis, and two target-vessel revascularization patients were reported within 1 year. Among these, myocardial infarction, nontarget-lesion revascularization, and in-stent thrombosis were reported within the first 30 days after discharge. These findings are per the findings of Abellas-Sequeiros et al. (3) reporting the safety and efficacy of magnesium-based BRS (Magmaris) with target lesion failure (TLF) of 4.7% at 12 months. The systematic review and meta-analysis by Wan et al. reported a significantly higher in-stent diameter stenosis of BRS compared with metallic DES within 1 year with a similar risk of target lesion revascularization and cardiac death. The metanalysis included 13 studies with 9,702 patients and compared the short- and mid-term outcomes between polymer- and magnesium-based BRS and newer-generation DES. Compared to DES a significantly higher rate of TLF [RR, 1.22, 95% CI (1.03, 1.44)] and TVMI [RR, 1.39, 95% CI (1.09, 1.76)] was observed in the BRS group. Nevertheless, it is noteworthy that there is only one study using Magmaris™ BRS included in this metanalysis. The result was mainly attributed to polymer-based BRS.

Another systematic review and meta-analysis of 4 randomized clinical trials including patients with acute myocardial infarction comparing BRS with newer-generation DES on clinical outcomes with at least 12 months of follow-up by Liu et al. suggested that BRS is as safe as DES in this high-risk setting. This was based on the findings from 803 participants showing a higher risk of the device-oriented composite endpoint (RR 1.62, 95% CI: 1.02–2.57, *P* = 0.04) and major adverse cardiac events (RR 1.77, 95% CI: 1.02–3.08, *P* = 0.04) without any significant differences in patient-oriented composite endpoint in patients treated with BRS compared to with patients treated with DES. Peng et al. reviewed the safety and efficacy of early-generation polymeric and metallic BRS compared to newer-generation DES with a focus on the clinical implications of BRS on scaffold thrombosis (ST) and concluded that higher-than-expected incidence of ST associated with BRS limits its use and DES remains the first choice in the majority of cases undergoing PCI. However, these limitations were mitigated if BRS was used in combination with dual antiplatelet therapy. The in-vitro study by Bjorkman et al. assessed the mechanical performance of the Angel™ iron-based bioresorbable scaffold (IBS) (LifeTech Scientific Corporation, China) during overdilation which is often required in the pediatric population. The study concluded that thin struts of the Angel IBS allow for a lower profile with increased maneuverability and its use with smaller sheaths.

Overall, these studies indicated mixed results favoring early-generation BRS for PCI with modified advanced implantation techniques, intravascular imaging guidance, and anti-platelet therapy. In addition, the limitations may also be mitigated by using novel materials and technologies (6). Ongoing RCTs with newest-generation thinner-struts BRS will determine whether BRS with improved designs may challenge the current dogma of DES for every patient and lesion and potentially reshape the future of PCI.
